# Quantifying the nuclear localization of fluorescently tagged proteins

**DOI:** 10.1093/bioadv/vbaf114

**Published:** 2025-05-12

**Authors:** Julien Hurbain, Pieter Rein ten Wolde, Peter S Swain

**Affiliations:** AMOLF, Amsterdam, 1098 XG, The Netherlands; School of Biological Sciences, The University of Edinburgh, Edinburgh, EH9 3BF, United Kingdom; AMOLF, Amsterdam, 1098 XG, The Netherlands; School of Biological Sciences, The University of Edinburgh, Edinburgh, EH9 3BF, United Kingdom

## Abstract

**Motivation:**

Cells are dynamic, continually responding to intra- and extracellular signals. Measuring the response to these signals in individual cells is challenging. Signal transduction is fast, but reporters for downstream gene expression are slow: fluorescent proteins must be expressed and mature. An alternative is to fluorescently tag and monitor the intracellular locations of transcription factors and other effectors. These proteins enter or exit the nucleus in minutes, after upstream signalling modifies their phosphorylation state. Although such approaches are increasingly popular, there is no consensus on how to quantify nuclear localization.

**Results:**

Using budding yeast, we developed a convolutional neural network that determines nuclear localization from fluorescence and, optionally, bright-field images. Focusing on changing extracellular glucose, we generated ground-truth data using strains with a transcription factor and a nuclear protein tagged with fluorescent markers. We showed that the neural network–based approach outperformed seven published methods, particularly when predicting single-cell time series, which are key to determining how cells respond. Collectively, our results are conclusive—using machine learning to automatically determine the appropriate image processing consistently outperforms ad hoc approaches. Adopting such methods promises to both improve the accuracy and, with transfer learning, the consistency of single-cell analyses.

**Availability and implementation:**

We performed our analysis in Python; code is available at https://git.ecdf.ed.ac.uk/v1jhurba/neunet-nucloc.git.

## 1 Introduction

**Figure 1.**
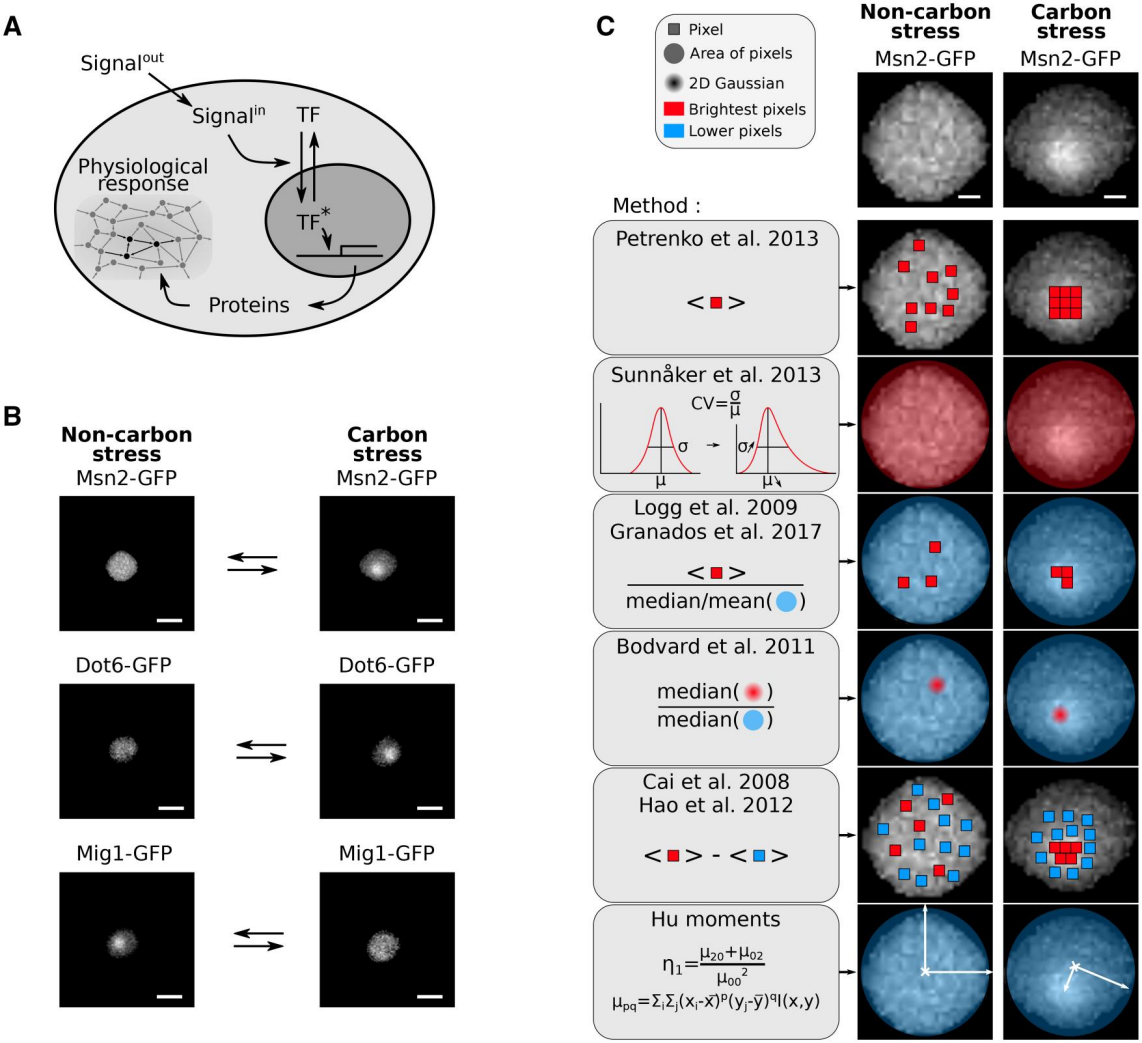
Detecting and quantifying the nuclear translocation of transcription factors. (A) A schematic showing a transcription factor entering the nucleus, regulating gene expression, and so cellular physiology through the proteins synthesized. $\mathrm{Signa}l^{\mathrm{out}}$ and $\mathrm{Signa}l^{in}$ are the extracellular and intracellular signals that generate active transcription factor. (B) The responses of three transcription factors in budding yeast to changes in extracellular glucose. Msn2 is the master regulator of the general stress response; Dot6 is a repressor of ribosome biogenesis; and Mig1 represses regulons for metabolizing alternative sugars to glucose. Scale bar = 4 µm. (C) Seven methods to quantify the localization of transcription factors. Left and right images show Msn2-GFP in a single cell. We indicate the pixels used by each method with colours, those with higher fluorescence in red and those with lower in blue. Areas and 2D Gaussians use ensembles of pixels. The first Hu moment, $\eta_{1}$, is a function of the pixels intensities at positions *x* and *y*. Scale bar = 1 µm. (B and C) Images in non-carbon stress are for 2% glucose; those in carbon stress are for 0% glucose.

Cells continually have to respond and adapt to changes in their environment. These changes are typically detected and relayed via signal transduction networks, which activate or deactivate transcription factors, leading to altered gene expression (Fig. 1A). In eukaryotes, changes in their activation state can cause the transcription factors to either enter or exit the nucleus (Vandromme *et al.* 1996). Examples in mammalian cells include NF-κB (Hoffmann *et al.* 2002, Tay *et al.* 2010) and p53 (Lahav *et al.* 2004) and at least tens of transcription factors translocate in budding yeast (Conrad *et al.* 2014).

Monitoring these transcription factors as they translocate is a versatile method to characterize how cells respond to changes in their environment or intracellular state. It can be applied to single cells, to time-varying environments, and to dynamic cellular responses and has a time resolution of minutes, much closer to that of the underlying signal transduction than reporters of gene expression.

For budding yeast, following the nuclear translocation of transcription factors and other proteins involved in regulating transcription has become a standard way to visualize and characterize stress responses over time. For example, the high-osmolarity glycerol (HOG) MAP kinase is often monitored to investigate hyper-osmotic stress (Hersen *et al.* 2008, Mettetal *et al.* 2008). Multiple transcription factors translocate in response to a fall in extracellular glucose: Msn2, a master regulator of the general stress response (Gasch *et al.* 2000, Causton *et al.* 2001), enters the nucleus (Jacquet *et al.* 2003, Hao *et al.* 2013) as does Dot6 (Gasch *et al.* 2017, Granados *et al.* 2018), a repressor of ribosome biogenesis (Lippman and Broach 2009), whereas Mig1 (Bendrioua *et al.* 2014, Lin *et al.* 2015), a repressor of regulons for metabolizing other sugars (Conrad *et al.* 2014), exits (Fig. 1B).

Although all the approaches developed use fluorescent-protein tags to identify transcription factors, they differ in how they quantify their intracellular localization (Fig. 1C). To avoid generating strains with a second fluorophore marking the nucleus, measuring the spatial homogeneity of the transcription factor’s fluorescence signal is often used as a proxy for nuclear localization—a transcription factor concentrated in the nucleus generates a more focused spot than one evenly distributed in the cytoplasm (Fig. 1B). Nevertheless, there is no consensus on how to measure spatial inhomogeneity, with at least seven different methods proposed for transcription factors in yeast alone (Table 1).

**Table 1.** Methods to quantify the nuclear localization of transcription factors from single-cell fluorescence images.

Name	Method	Reference
Petrenko	Mean intensity of the brightest 20% of pixels	Petrenko *et al.* (2013)
Sunnåker	Coefficient of variation of the pixel intensities	Sunnåker *et al.* (2013)
Logg	Ratio of the mean of the three brightest pixels to the mean of the remaining pixels	Logg *et al.* (2009)
Granados	Ratio of the mean of the five brightest pixels to the median of the remaining pixels	Granados *et al.* (2018)
Bodvard	Ratio of the median value of a smoothed Gaussian centred on the brightest pixel over the median of the remaining pixels	Bodvard *et al.* (2011)
Cai Hao	Difference of the mean of the five brightest pixels and the mean of the remaining pixels	Cai *et al.* (2008) Hao and O’Shea (2011)
Hu moments	First invariant moment $\eta_{1}$	Hu (1962) Nasrudin *et al.* (2021)
Neural network	Prediction by a convolutional neural network	This study

Noting their continuous adoption (Bergen *et al.* 2022, Jonas *et al.* 2023, Hong *et al.* 2024, Polisetty *et al.* 2025), we set out to evaluate the different methods, comparing with one that we developed here using a convolutional neural network (CNN) with an everyday architecture. We included too an older standard for measuring spatial inhomogeneities: Hu moments (Hu 1962, Nasrudin *et al.* 2021), which use the moments of inertia treating the fluorescence values within a cell as masses. We found that our neural network–based method outperformed the others.

The purpose of our paper is therefore to demonstrate that a simple neural network—one requiring little training data or computational resource and little effort to set up—can outperform the established measures of nuclear localization. Switching to using this network should be straightforward for most microscopy laboratories and give substantial increases in accuracy. As we show, the training data can be generated locally in a single experiment using strains with nuclear markers.

## 2 Results

### 2.1 Finding transcription-factor localization from a nuclear marker

**Figure 2.**
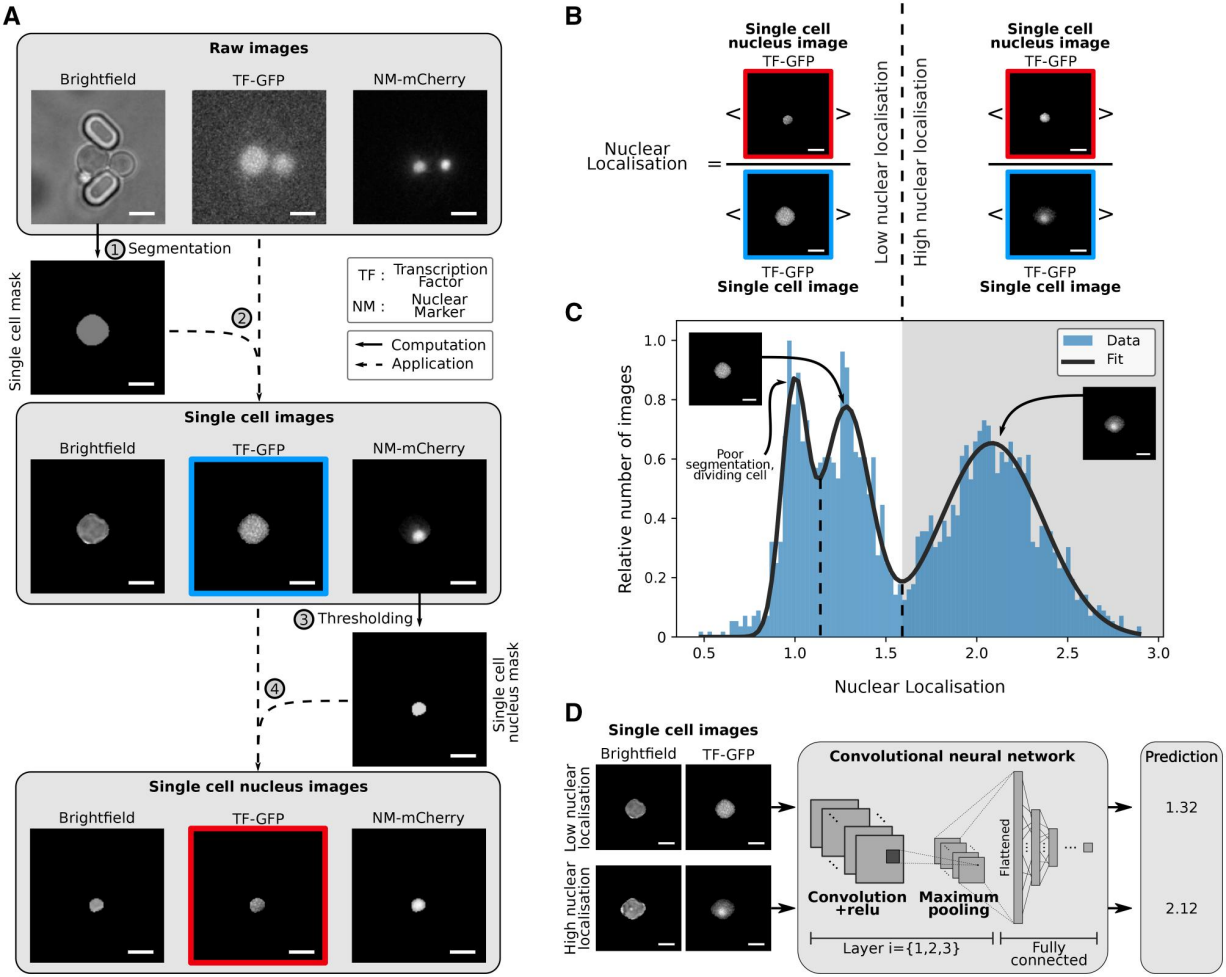
Estimating the nuclear localization and preparing images for analysis by a neural network. Scale bar = 4 µm. (A) Our pipeline for preparing images for analysis. Cells grow stably between two pillars in the ALCATRAS microfluidic device (Crane *et al.* 2014), which has hundreds of these traps. Using the BABY algorithm (Pietsch *et al.* 2023), we segment single cells from the bright-field images and find the pixels corresponding to these cells in the fluorescence images. With Otsu thresholding of the image of the nuclear protein Nhp6a-mCherry, we determine the pixels in the nucleus. For each cell, we therefore have a bright-field image, a fluorescence image showing the intracellular fluorescence from a tagged transcription factor, and a binary image identifying the nuclear pixels. (B) A schematic showing the definition of nuclear localization [Equation (1)]. (C) The distribution of nuclear localization typically has three peaks. We show the distribution for Msn2-GFP, combining images from a time-lapse experiment where we switched extracellular glucose from 2% to 0%. The images are from just before and after the switch. (D) The convolutional neural network has three layers and predicts nuclear localization using the maximum projection over Z stacks of both fluorescence and bright-field images as inputs.

We generated time-lapse single-cell data with both a fluorescent transcription factor, tagged with green fluorescent protein (GFP), and a fluorescent nuclear marker, the Nhp6a gene tagged with mCherry (Hansen *et al.* 2015, Granados *et al.* 2018) (Fig. 2A). The Nhp6a protein remodels nucleosomes (Ruone *et al.* 2003). We used the ALCATRAS microfluidic device to grow and trap cells (Crane *et al.* 2014) and the BABY algorithm (Pietsch *et al.* 2023) to segment and track these cells over time from time-lapse, bright-field images.

To identify the nucleus from the mCherry images, we used Otsu thresholding (Otsu 1979). Just as there is no consensus on the optimal way to measure the localization of transcription factors, there is none too on how to determine the nucleus’s location (Jacquet *et al.* 2003, Li *et al.* 2018, Duveau *et al.* 2024). In contrast to transcription factors, however, the fluorescence from a nuclear marker is consistently bright over both time and extracellular conditions (Fig. 1C), and we found little difference between most methods (Supplementary Fig. S1), all of which threshold the image in some way (Johannsen and Bille 1982, Kapur *et al.* 1985, Bernsen 1986, Parker 2010). We chose Otsu thresholding because it has no parameters; it does though require a bimodal distribution of pixel fluorescence intensities (Goh *et al.* 2018) and so a sufficiently bright nuclear marker. These conditions were satisfied in our experiments.

With the nucleus identified, we defined, following (Duveau *et al.* 2024), the degree of nuclear localization as


(1) $$\ell =\frac{\langle I_{\text{nuc}}\rangle }{\langle I_{\text{cell}}\rangle }$$

where $I_{\text{nuc}}$ represents the pixel intensities in the nucleus and $I_{\text{cell}}$ those for the whole cell, including the nucleus (Fig. 2B). Often researchers wish to predict the likelihood of downstream gene expression for which the most relevant quantity is the nuclear concentration $\langle I_{\text{nuc}}\rangle$, the total fluorescence in the nucleus divided by its area. We can recover this concentration from the localization by multiplying Equation (1) by $\langle I_{\text{cell}}\rangle$, the mean cellular fluorescence, which is commonly measured.

Applying our workflow to cells in different carbon sources, we typically found a tri-modal distribution of the level of nuclear localization (Fig. 2C). The largest mode corresponds to cells with a high nuclear concentration of transcription factor; the middle node to cells with a low nuclear concentration; and the lowest mode, based on visual inspection of the images, either to poorly segmented cells, such as those overlapping the edges of an image, or to cells actively dividing with consequently an imperfectly identified nucleus. We therefore consider cells with predominantly cytoplasmic transcription factors to have 1.15<ℓ<1.65 and cells with predominantly nuclear transcription factors to have ℓ>1.65.

### 2.2 A CNN to predict nuclear localization

We developed a CNN (Goodfellow *et al.* 2016) to predict nuclear localization from segmented fluorescence images of tagged transcription factors (Fig. 2D). The network comprises three layers and a fully connected layer (Supplementary Fig. S2A) and maps microscopy images of a single cell to one continuous value, the predicted level of nuclear localization. As inputs, we used the maximum projection of fluorescence images taken at five Z sections and, although not required, the maximum projection of the corresponding five bright-field images. Such bright-field images are readily available and improved accuracy because the nucleus can sometimes be partly seen in bright-field (Adjavon 2022). We used the nuclear Nhp6a-mCherry marker only to establish a ground-truth data for training; the corresponding images were not used as network inputs. After training, using data from the transcription factors Msn2, Dot6, and Mig1, the network had an accuracy of 95% (Supplementary Fig. S2B). Excluding data from one specific transcription factor while training but not while testing neither substantially nor consistently affected performance (Supplementary Fig. S2C). For instance, removing data from Msn2 while training does not always lead to poor performance while testing with that data.

### 2.3 Comparing different methods

**Figure 3.**
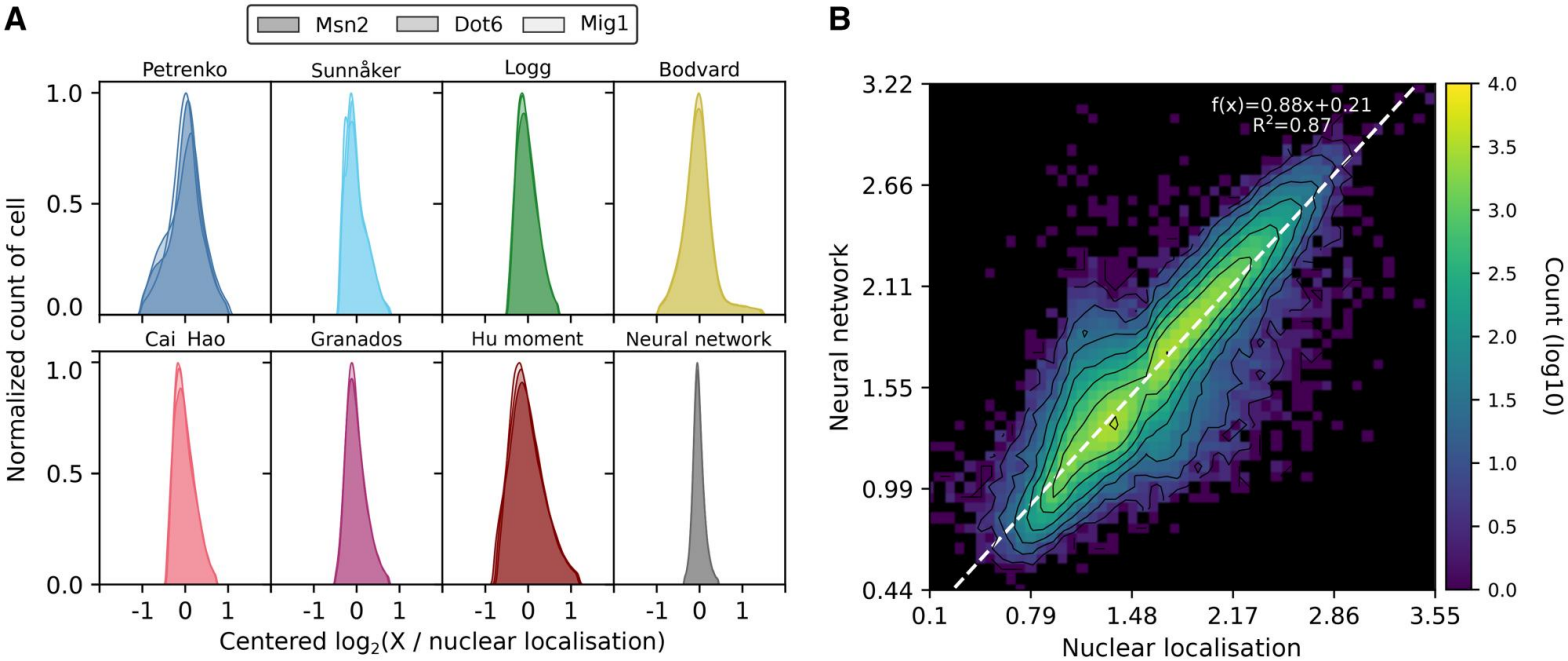
The neural-network approach best identifies nuclear localization from among the different methods. (A) The neural network has the tightest distribution of errors when comparing predictions to the ground-truth level of nuclear localization found from the Nhp6A-mCherry marker. Data are for Msn2-GFP, Dot6-GFP, and Mig1-GFP from cells in a step from 1% to 0% extracellular glucose. We plot the ${\;\text{log}\;}_{2}$ of the predicted to the ground-truth value and subtracted the mean over all cells, so that each method has an identical mean of zero. For the approach of Bodvard *et al.* (2011), we ensured all values are positive by incrementing each by one. (B) Plotting the neural network’s prediction against the ground-truth level of nuclear localization shows a tight linear relationship. The shading indicates numbers of cells on a $log_{10}$ scale, with zero cells in black. The corresponding plots for the other methods are in Supplementary Fig. S4.

With the nuclear marker determining the ground-truth level of nuclear localization, we compared the various methods using three transcription factors that respond to extracellular glucose (Fig. 3A). Two of these enter the nucleus in low glucose while the other exits (Fig. 1B). A difficulty is that each method makes predictions over a different numerical range because each has its own way of characterizing spatial inhomogeneity. We therefore plotted the log_2_ ratio of the method’s predicted value to the ground-truth level of nuclear localization and centred the results by subtracting each method’s mean ratio (non-centred results are in Supplementary Fig. S3). Accurate methods should have a tight, symmetric distribution.

The neural network performed best. Although having a skewness comparable to other approaches (Supplementary Fig. S3), it had the lowest standard deviation and highest consistency over the three transcription factors. Plotting the predicted localization versus the ground truth (Fig. 3B), the neural network’s results tightly followed the y=x line (Supplementary Fig. S4), and both its correlation and mutual information with the ground truth were highest (Supplementary Fig. S5). Unlike the other methods, as it is the only one trained to do so, we can also directly interpret its predictions as estimates of Equation (1).

Although the neural network’s performance is perhaps unsurprising, being the only method benefiting from the training data, it is useful to quantify how much better it is over the alternatives. To this end, we investigated time-series—the focus of multiple studies (Cai *et al.* 2008, Hersen *et al.* 2008, Mettetal *et al.* 2008, Hansen and O’Shea 2013, Hao *et al.* 2013, Bendrioua *et al.* 2014, Lin *et al.* 2015, Goulev *et al.* 2017, Granados *et al.* 2017, 2018, Pietsch *et al.* 2023, Duveau *et al.* 2024). The results further highlight how much our CNN-based method can improve the prediction of nuclear localization.

### 2.4 The neural network’s predictions best capture dynamic single-cell responses

**Figure 4.**
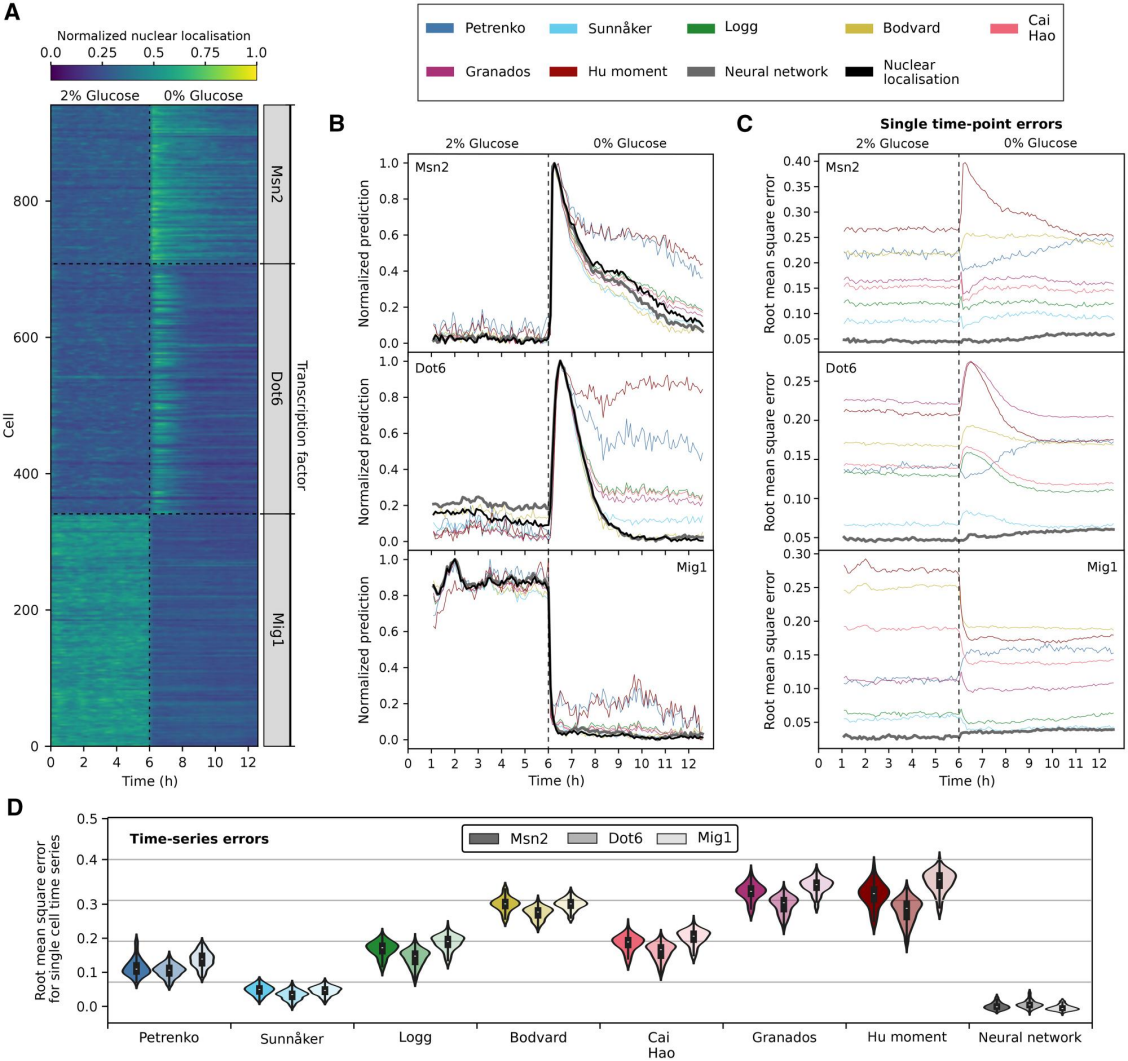
The neural network best predicts single-cell time-series of nuclear localization. (A) The ground-truth time series of nuclear localization for cells experiencing a drop in glucose from 1% to 0% after 6 h of growth. Each row represents a single cell with the shading showing the normalized degree of nuclear localization. For Msn2, n=233; for Dot6, n=360; for Mig1, n=341. (B) The predicted mean level of nuclear localization over all cells at each time point varies between the different methods (predictions for individual cells are in Supplementary Fig. S6). The grey curve corresponding to the neural network is so close to the ground truth that it is often obscured. We normalized each prediction to be between zero and one. For Petrenko *et al.*’s (2013) method, we inverted their prediction so that it behaves similarly to the others. (C and D) The root mean square error is defined as the root mean square of the difference between the ground-truth nuclear localization and that identified by the method. (C) The root mean square errors at each time point for all cells. (D) The distributions over all cells of the root mean square errors for predicting entire time series. For each cell, we calculated the root mean square error based on the predictions made at every time point and then repeated this process for all cells to generate a distribution of root mean square errors. Results for Msn2 are on the left and for Mig1 on the right.

Information transmission, and more broadly, the variation in how individual cells respond to changes in their environment, often manifests not in the response at a given moment in time nor in the long-time limit, but rather in the full dynamical trajectory of the response over time (Tostevin and Ten Wolde 2009, Granados *et al.* 2018). In constant glucose (Fig. 4), both Msn2 and Dot6 stochastically enter and exit the nucleus in some cells (Dalal *et al.* 2014), impeding straightforward averaging, which would obscure this behaviour. Almost all cells responded, however, when we removed extracellular glucose, with Msn2 entering the nucleus on average faster than Dot6.

Our data are a time series of images taken every 5 min. Processing these images into time series for each cell (Fig. 4A) and applying the methods time point by time point for each time series, we saw substantial differences even in predicting the mean nuclear translocation (Fig. 4B). Although all methods identified that the cells responded to the drop in extracellular glucose, shown by a peak in the time series, some had difficulty when the transcription factors were predominantly in the nucleus, with higher errors for Msn2 and Dot6 after glucose drops and for Mig1 before. Only the neural network’s predictions had low errors both at each time point (Fig. 4C) and averaged over all the time points in each single-cell time series (Fig. 4D).

## 3 Discussion

Our study shows that machine learning, through the CNN we developed, consistently outperforms *ad hoc* approaches, particularly for single-cell time series. Such time series best characterize individual responses (Murugan *et al.* 2021).

We opted for a CNN because this architecture balances the trade-off between accuracy versus ease of implementation and training. Although a more complex architecture such as a visual transformer (Liu *et al.* 2024) likely can increase accuracy, such networks require more training data and more computational resources having an order of magnitude more parameters.

Although neural networks require training data, so too do *ad hoc* approaches because without such data these approaches cannot be validated. The difference lies in the quantity of training data, but with microfluidic technology, generating sufficient data is possible in a single experiment, as we demonstrated here. Additionally, the quality of the approach can be continually improved by adding more images to the training data, such as from different microscopes, imaging protocols, strains, or species. Even better, a network trained in one laboratory will likely need much less data to be re-trained for another, with the promise to improve the consistency of signal-cell analyses broadly.

Single-cell data are often noisy, and a poor choice of signal can further reduce the signal-to-noise ratio, confounding analyses. For nuclear translocations at least, we have shown that using machine learning to identify the signal, through optimizing the image processing required, is one way to gain accuracy and with it, no doubt, greater biological insight for the future.

## 4 Methods

### 4.1 Strains

Details of all strains are given in Granados *et al.* (2018) [Synthetic Complete (SC)] medium at 30°C either supplemented with either 2% or 1% glucose or with no additional carbon source, denoted 0% glucose.

### 4.2 Cell preparation and loading ALCATRAS

We used overnight cultures in a 30°C incubator under agitation to generate mid-log cells, which we diluted in fresh SC medium to an optical density of 0.1 and then incubated for a further 3 h before loading into a microfluidic device. To expose different strains to the same extracellular conditions, we used a multi-chamber version of ALCATRAS (Crane *et al.* 2014). Prior to loading, the ALCATRAS chambers were pre-filled with growth medium supplemented with 0.05% bovine serum albumin (BSA) to facilitate cell loading and reduce cell clumping.

### 4.3 Time-lapse microscopy

Our microscope is a Nikon Ti Eclipse, optimized for imaging GFP, mCherry, and Cy5 fluorescence, as well as bright-field transmission. For GFP, we used a Chroma dual-band filter set (59 022) with an excitation range of 452–490 nm, centred at 470 nm, a beam splitter range of 496–548 nm, and an emission filter at 535/30 nm (520–550 nm range). The mCherry imaging shared the dual-band filter set for GFP, with an excitation range of 554–558 nm (centred at 556 nm), a beam splitter range of 595–677 nm, and an emission filter at 632/60 nm. Finally, for Cy5 imaging, we used an excitation filter at 620/60 nm (590–650 nm, centred at 620 nm), a 660 nm longpass dichroic, and a 665 nm longpass emission filter. For Cy5 and bright-field transmission, the microscope had a white LED with a broad-spectrum range, and for fluorescence imaging, an OptoLED light source from Cairn Research.

The objective was a Nikon 60× oil-immersion lens with a numerical aperture (NA) of 1.4, using Nikon F2 immersion oil. Imaging was captured with a Teledyne Prime 95 b sCMOS camera, with an 11 µm pixel size, 16-bit dynamic range, 1 × 1 binning, gain of 1, and air cooling at −15°C. Exposure time for imaging was set at 30 ms, with images captured every 5 min for 150 time points, i.e., 12.5 h. We acquired bright-field and fluorescence images at five Z-sections spaced by 0.6 µm, but in a single focal plane for the Cy5 channel. Both the microfluidic device and the media were kept at 30°C inside an incubation chamber (Okolabs). Nikon’s Perfect Focus System maintained consistent focus.

We used Fluigent pressure-driven system to control the flow of media and to switch extracellular glucose concentrations. We applied carbon stress after 6 h by switching the flow rate of either 2% or 1% glucose medium from 9 µL/min to 1 µL/min and the flow rate of 0% glucose medium from 1 µL/min to 9 µL/min. Slowly flowing medium did not enter the chamber containing cells and was redirected to waste. We used Cy5 dye in the 0% glucose medium to confirm media switches. Media were supplemented with 0.05% of BSA to facilitate cell loading and reduce cell clumping.

### 4.4 Image segmentation

To segment and track cells, we used BABY (Pietsch *et al.* 2023) and the aliby Python pipeline (Muñoz González 2023).

To identify nuclear pixels, we used Otsu thresholding on single-cell images after applying a Gaussian blur (Gonzalez and Woods 2009). Alternative methods, which we also tested, are in Table 2.

**Table 2.** Image-processing methods to identify pixels in the nucleus from fluorescence images of a nuclear marker.

Name	Method	Reference
Ostu	Global thresholding	Otsu (1979)
Kapur	Entropy thresholding	Kapur *et al.* (1985)
Johannsen	Entropy thresholding	Johannsen and Bille (1982)
Bernsen	Local thresholding	Bernsen (1986)
Contrast	Local thresholding	Parker (2010)

We estimated mutual information with the nearest-neighbour approach (Kraskov *et al.* 2004, Ross 2014).

### 4.5 Constructing and training the neural network

We optimized, using random sampling, both the hyperparameters and the architecture of the neural network using a subset of the training data of approximately 12 000 images. The training hyperparameters were the batch size (from 16 to 256), the total number of epochs (from 20 to 200), the learning rate (from $10^{-1}$ to $10^{-5}$), the number of epochs for the learning-rate scheduler (from 20 to 100), and the optimization algorithm (Adam or stochastic gradient descent). The hyperparameters determining the network architecture were the number of input channels (from 4 to 32) in the first convolution layer with the number doubling in subsequent layers, the total number of convolution layers (from 1 to 3), the number of convolutions for each layer (from 1 to 3), whether the last convolution layer either increases the number of channels and uses maximum pooling or keeps the number of channels fixed, and the number of fully-connected layers (from 1 to 3). We selected hyperparameters by comparing accuracy, defining a cell to have a localized transcription factor if their nuclear localization, Equation (1), was above 1.65. The optimization suggests that there is no unique best architecture, but a diverse set of well-performing ones. Using the Adam optimizer with both a small batch size and learning rate, however, was strongly favoured.

The network we used had three convolutional layers (Supplementary Fig. S2A). The first two layers are followed by Relu activation functions and maximum pooling. The third layer has no maximum pooling, and we flatten its output into a one-dimensional array. The final, fully-connected layer is a linear transformation with a ReLu function followed by another linear transformation to a scalar value.

For training (Supplementary Fig. S2B), we used mean square error as the loss function and the Adam optimizer. We used a batch size of 64, a CUDA implementation of the network for speed (Garland *et al.* 2008), and a learning rate of $10^{-3}$ with a linear learning-rate scheduler of ratio 0.5 over 50 epochs.

The training data comprised around 180 000 single-cell images with 90% randomly selected for training and 10% for validation. We used data from an experiment with a strain with MSN2-GFP and a switch of extracellular glucose from 1% to 0%. To test the network, we used an entirely different data set also with MSN2-GFP but a switch of glucose from 2% to 0% (Figs 3–4 and Supplementary Figs S3–S6).

For coding, we used Python and Pytorch with the CUDA package.

## Supplementary Material

vbaf114_Supplementary_Data

## Data Availability

Code and sample data used in this work can be found at https://git.ecdf.ed.ac.uk/v1jhurba/neunet-nucloc.git. The full dataset set with its corresponding code is available at https://doi.org/10.7488/ds/7820.
