# Supplementary material for: Quantifying the nuclear localization of fluorescently tagged proteins
Source: Bioinform Adv. 2025 May 12;5(1):vbaf114. doi: 10.1093/bioadv/vbaf114 (PMC12133273; doi:10.1093/bioadv/vbaf114)
Supplement: vbaf114_Supplementary_Data [file vbaf114_supplementary_data.pdf]

Supplementary information: Quantifying the nuclear localisation of fluorescently tagged proteins

Julien Hurbain[1,2], Pieter Rein ten Wolde[1], and Peter S. Swain[2]

[1]AMOLF, The Netherlands
[2]School of Biological Sciences, University of Edinburgh

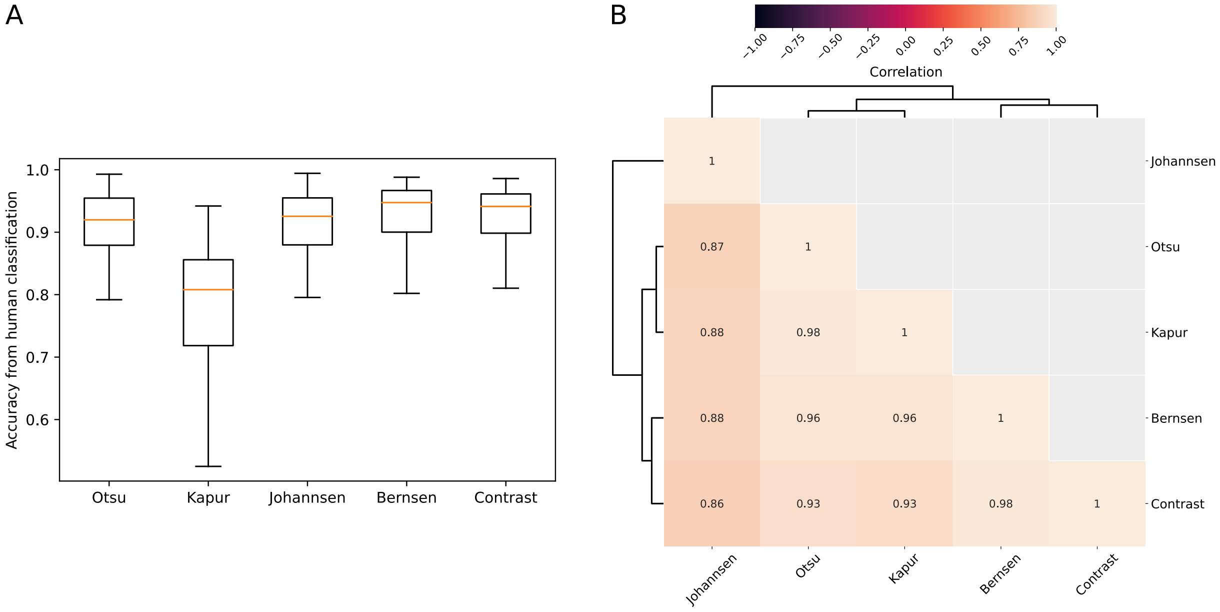

Figure S1. A comparison of different methods to identify the nucleus. (A) A comparison of the different methods for identifying the nucleus from a fluorescence image of a nuclear marker (Table 2). To establish a ground truth, we identified the nucleus by eye in approximately 200 images. (B) A matrix showing the high correlation between the different methods.

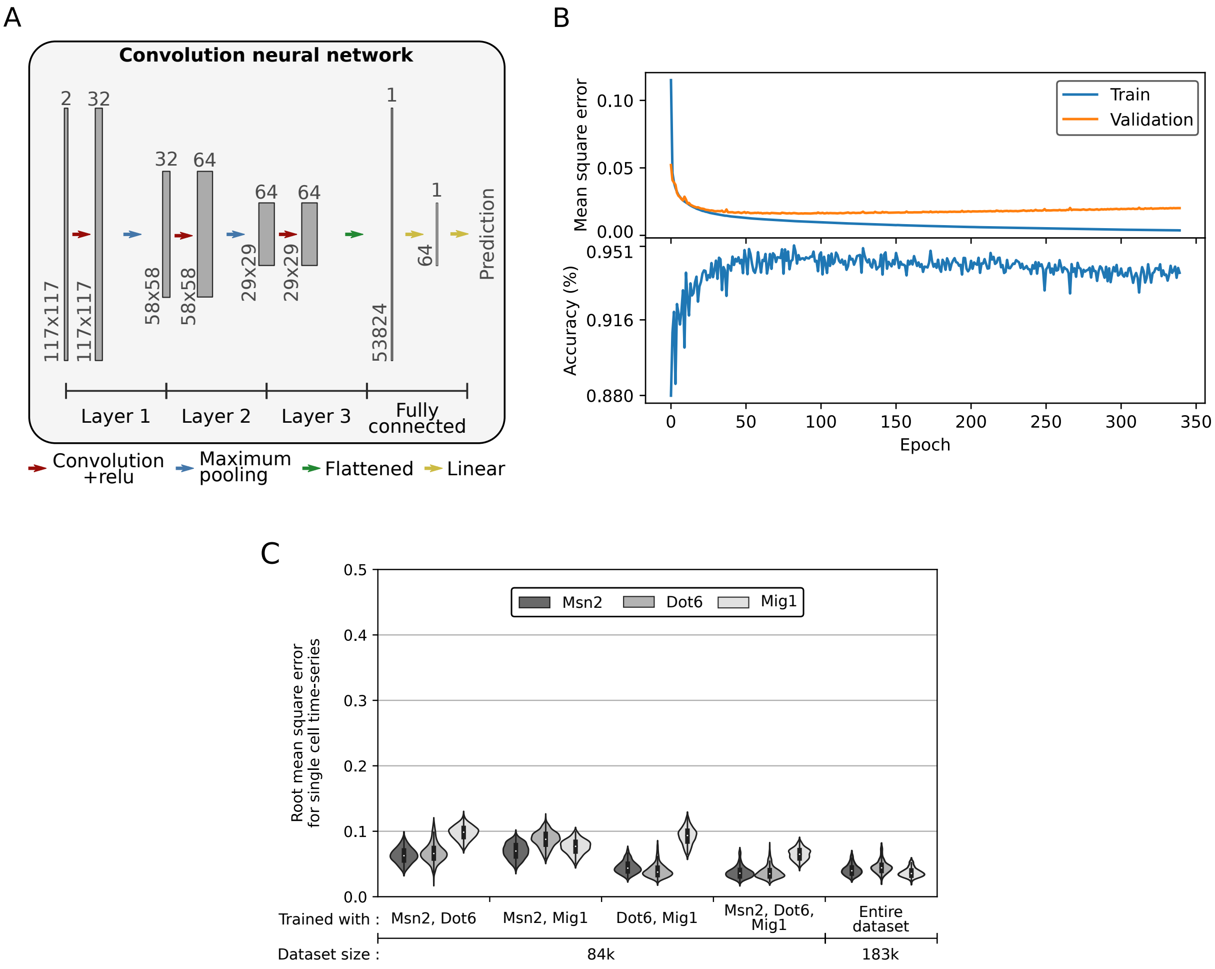

Figure S2. A neural network to predict localisation from fluorescence and, optionally, bright-field images. (A) The structure of the convolutional neural network. Each gray rectangle represents a different stage as the network processes the image. The size of the image is indicated on the side and the number of feature channels on top. The single-cell images we use as inputs are 117×117 pixels and have two channels: fluorescence and bright-field. (B) Typical training curves for the network. We used a mean-square loss function and $\approx 180,000$ images with 90% randomly selected for training and 10% for validation. To compute an accuracy, we applied a threshold to the predicted level of nuclear localisation (the threshold divides the white from the gray shaded areas in Fig. 2C). (C) The distributions of root mean square errors for each single-cell time series of the dataset used in Fig. 4 for networks trained on data from different pairs of transcription factors ignoring one. We chose the number of images to allow equally sized datasets for all the training conditions. The results from the entire dataset are the same as Fig. 4D. We show Msn2 are on the left and Mig1 on the right.

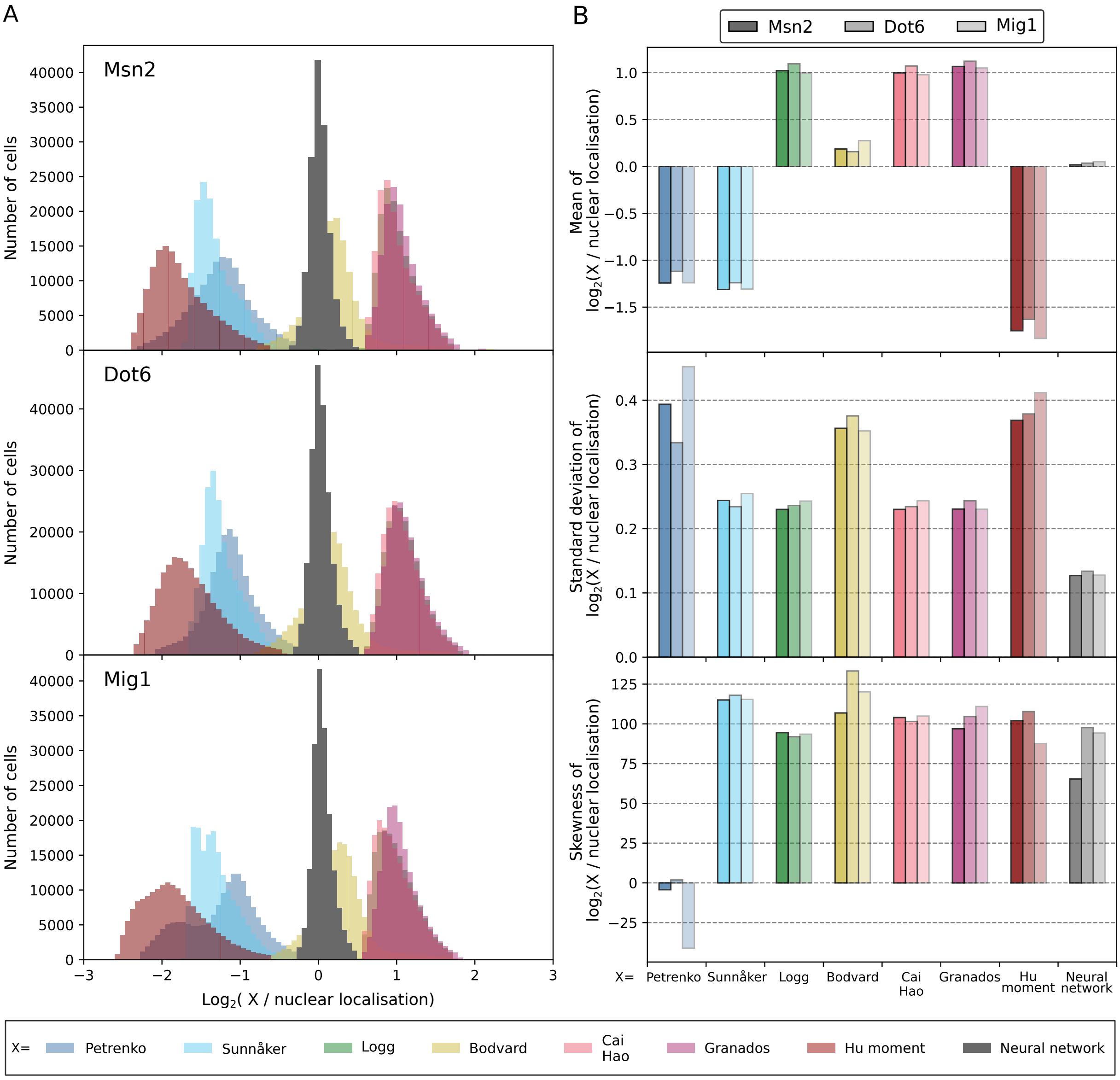

Figure S3. Comparing predictions of nuclear localisation on an absolute rather than relative scale. (A) The error distributions for the different methods of predicting nuclear localisation from Table 2. Unlike in Fig. 3A, these distributions are not normalised to have zero mean. Most poorly predict Eq. 1 because they are not explicitly designed to do so, although their values do correlate with the transcription factor's localisation. We have combined data for Msn2-GFP, Dot6-GFP, and Mig1-GFP in a step from 1% to 0% extracellular glucose at all time points. For the Bodvard *et al.* method [1], we ensured values are always positive by incrementing each value by one. (B) The mean, standard deviation, and skewness of the error distributions. We use shading to indicate the different transcription factors, with Msn2-GFP on the left and Mig1-GFP on the right.

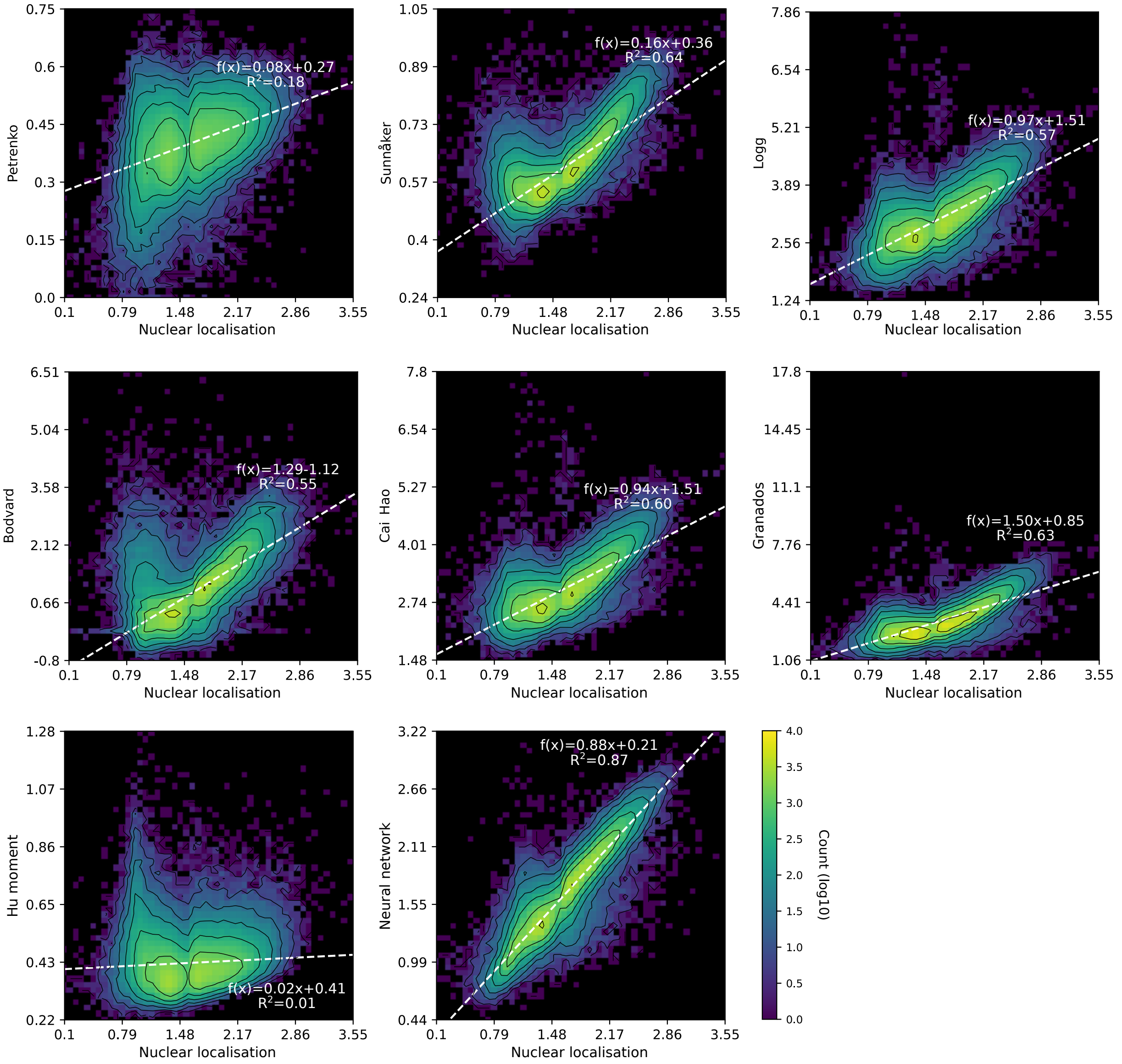

Figure S4. The predicted nuclear localisation versus the ground-truth localisation. Colours represent the number of cells on a $\log_{10}$ scale; black represents no cells. The white dashed-line is a linear regression. Ideal predictions should be linearly proportional to the ground truth, with a high R^2.

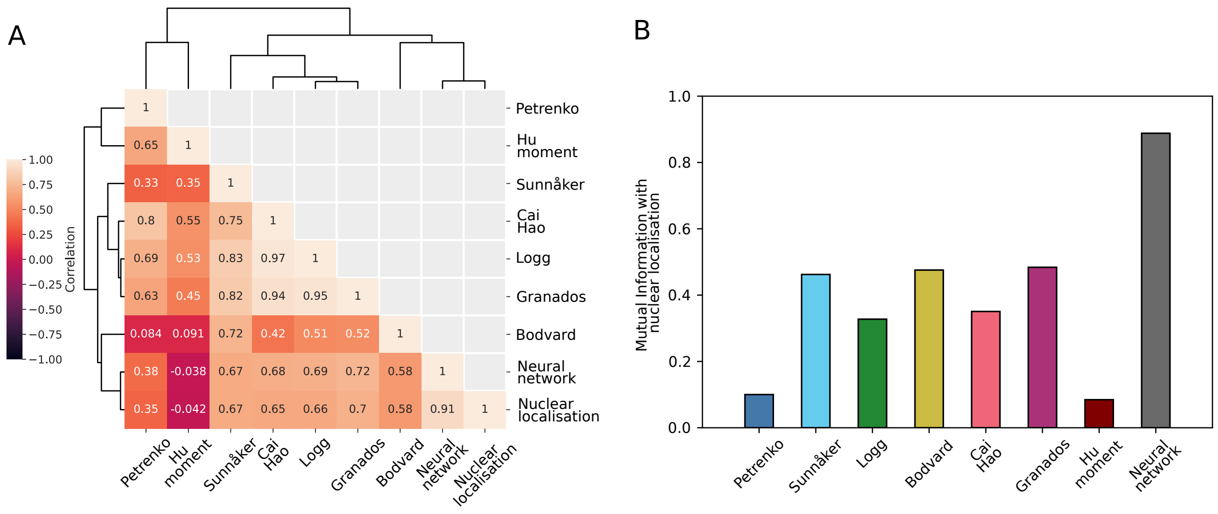

Figure S5. Statistically comparing the methods' predictions to the ground-truth localisation favours the neural network. (A) A correlation matrix of the methods' predictions. Only the magnitude of the correlation is relevant because some methods are anti-correlated with the ground truth. (B) The mutual information between the predictions and the ground-truth localisation. Unlike the correlation coefficient, the mutual information measures both linear and non-linear relationships between two variables.

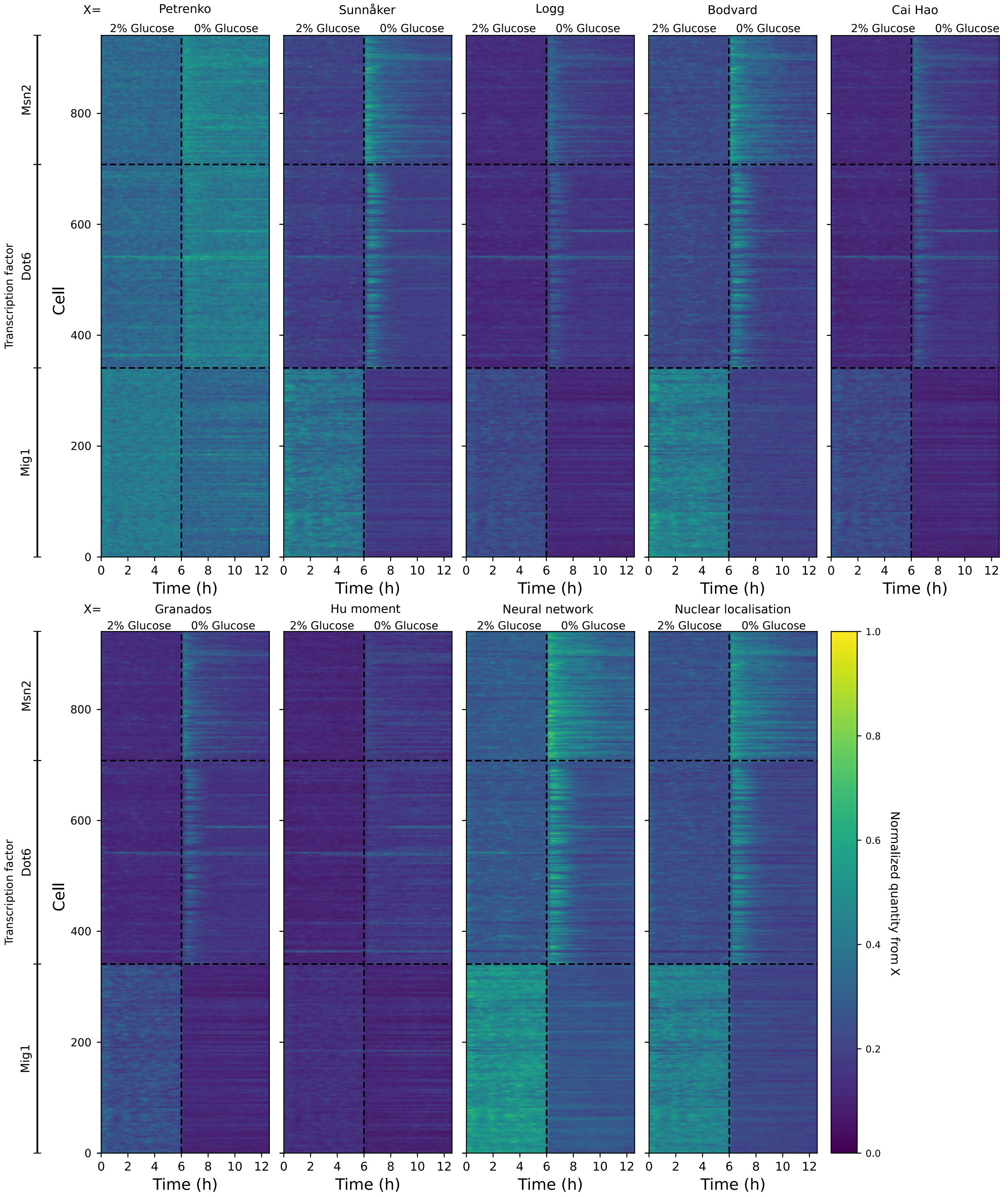

Figure S6. Predictions of nuclear localisation for individual cells during a drop in extracellular glucose. Each row represents a single cell, with the colour code showing the predicted level of nuclear localisation normalised to be between zero and one. We decreased the extracellular glucose concentration from 1% to 0% after six hours. To aid comparison, we inverted the values predicted by Petrenko *et al.*'s method [2], subtracting their prediction from one.

References

1. Bodvard, K. *et al.* Continuous light exposure causes cumulative stress that affects the localization oscillation dynamics of the transcription factor Msn2p. *Biochim Biophys Acta, Mol Cell Res* **1813,** 358–366 (2011).
2. Petrenko, N., Chereji, R. V., McClean, M. N., Morozov, A. V. & Broach, J. R. Noise and interlocking signaling pathways promote distinct transcription factor dynamics in response to different stresses. *Mol Biol Cell* **24,** 2045–2057 (2013).
